# Memorial of Dr. T. S. Powell

**Published:** 1896-04

**Authors:** J. McFadden Gaston

**Affiliations:** Professor Principles and Practice of Surgery. Presented at the Seventeenth Commencement Exercises, Southern Medical College; Atlanta, Ga.


### Southern Medical Record.

A MONTHLY JOURNAL OF MEDICINE AND SURGERY.

VOL. XXVI. ATLANTA, GA., APRIL, 1896. No. 4.

### Original Articles.

### MEMORIAL OF DR. T. S. POWELL.

BY DR. J. MCFADDEN GASTON,
Professor Principles and Practice of Surgery.

Presented at the Seventeenth Commencement Exercises, Southern Medical College, March 30, 1896.

When called upon short notice to make some remarks before those assembled here a year ago, in place of the President of the Southern Medical College, little did I think it would devolve upon me to note on this occasion, his departure from this field of activity to receive his reward in a higher and better sphere of spiritual existence.

After fifty years of intensive devotion to the work of humanity in the medical profession and with the consciousness of a well spent life, he realized that his heart's desire was fulfilled in the accomplishment of those ends for which he had labored so faithfully. It was a most pathetic and impressive calm which spread over his being during the last days of his illness, when compared with the energetic and incessant struggle of long continued effort in the performance of duty.

The intellectual activities and benevolent impulses which ennobled the life of Dr Thomas S. Powell were stilled by death on December 30, 1895. His last day prior to the fatal attack, was spent in lecturing to the class and in visiting patients, so that he died at the post of duty.

The unceasing interest with which his best efforts were devoted to the advancement of medical education, was a labor of love with him, and his undertakings were never marked by any failures to accomplish his object.

The prudent forecast and zealous energy manifested by Dr. Powell in the welfare of the Southern Medical College during the last sixteen years, as its founder and president, were fully appreciated by the faculty, who heartily endorsed the general recognition of his valuable services to the institution.

The present high standing of the Southern Medical College is due chiefly to the wise administration by the president, and it should stand forth as the noblest monument which could be erected to his honor and good name.

It was with the greatest sorrow for the loss of our faithful counselor and true friend, that the faculty of the Southern Medical College set apart a page in the book of their proceedings to record the lamented death of our president. The mingled feeling of interest on the part of professors and students of the college made it fit and proper to dispense with the regular duties of the lecture rooms on the day following the death of Dr. Powell, and a joint meeting was held for presenting suitable tributes to his memory.

The faculty tendered its most profound sympathy to her who had so nobly shared the cares and responsibilities of her husband's honorable career in life, trusting that Heaven's choicest blessings should attend her future days on earth.

The college building was draped in mourning as a mark of our bereavement and high consideration for our president.

Resolutions of respect and sympathy were passed by the trustees, students, and the Alumni Association, of the Southern Medical College, independently of this action of the faculty.

Dr. Powell's funeral occurred at Trinity Church, Jan. 1, 1896, when eulogies were made upon his exemplary Christian life, and his body was interred at Sparta, Ga. The body of Dr. Powell's first wife, and the remains of his parents and child are there mingling with his. The many marks of appreciation shown during his illness and death, amply testified to his place in the hearts of all.

The faculty presented a beautiful wreath of flowers, while the Alumni Association of the Southern Medical College also showed their sympathy for his love of music by a large harp of violets, hyacinths and roses, resting upon a pillow. Truly in the words of the lamented poet laureate:

> "O good gray-head which all men knew,
> O voice from which their omens all men drew,
> O iron nerve, to true occasion true;
> O fallen at length that tower of strength,
> Which stood foursquare to all the winds that blew."

In connection with the obituary notice of Dr. Powell, it is becoming to give some details of his career in life, taken from the records found in various publications, not accessible to the general reader, the data being furnished to a large extent by the subject of this sketch.

While this information is appropriated from different sources, without being directly credited, I wish in advance to acknowledge my obligation to others for valuable matter, and in many instances, for the language used in these communications.

It is hoped that thus I may not be liable to the charge of literary piracy or plagiarism in presenting these points culled from current literature in preparing this brief memoir.

Dr. Thomas Spencer Powell, who was of Welsh descent, was born in Brunswick County, Virginia, October 5, 1824. His collegiate education was commenced at Oakland Academy, in his native State, under Prof. J. P. Atkinson, the father of our present governor, and completed with honor at Lawrenceville Male Institute, then in charge of the celebrated Professor Brown, of William and Mary College. Even in early childhood, he manifested such a predisposition for the practice of the healing art, by playing doctor in treating the little negroes of the plantation with bread pills, etc., that his father accepted it as a suggestion, and decided that medicine should be his profession.

His education, therefore, was directed with special reference to this purpose, and when his collegiate course had ended his father placed him under the care of Dr. Benjamin I. Hicks, of Lawrenceville, Virginia. Dr. Powell was taught practical pharmacy in the manufacture and compounding of drugs for his preceptor, a custom of that period which was far preferable to the methods of the present.

After two years of preparatory reading and training, he attended two full courses of lectures at the Medical Department of the University of Pennsylvania, graduating with great distinction in the spring of 1846, having received a higher vote than any other member of his class. In September, 1846, he located in Sparta, Georgia, where he announced himself as a practicing physician. Prompt in responding to professional calls; kind, charitable and courteous to all; eminently successful in the treatment of his patients, he soon commanded an extensive and lucrative practice.

In 1847 he married Miss Julia L. Bass, daughter of Rev. Larkin Bass and granddaughter of Governor Rabun, of Georgia, a highly educated and accomplished lady, beloved by all who knew her, for her charming character. She died, June 10, 1880. Their only child died as a little girl of four years. Dr. Powell married again December 28, 1882. His second wife was Mrs. Jennie Miller, of Virginia, a lineal descendant of a renowned Scotch family, Roxboro, from whom the town of Roxborough, Scotland, derived its name. She survives him.

Dr. Powell was not only a successful physician, but an enterprising and public-spirited citizen, quick to observe the wants of his section and resolute in enforcing such enterprises as tend to promote the general welfare.

In the early period of his practice, he wrote and published a medical work known as the "Pocket Formulary and Physician's Manual," which was most favorably noticed by the journals of that day and highly appreciated by the members of his profession.

In 1857, he was invited to deliver the annual address to the graduating class of the Atlanta Medical College, his subject being: "The Moral Duties of the Physician." The address was most favorably received. He handled his subject with great ability and eloquence, so impressing his audience and the faculty of the college that he was subsequently elected to an important chair in that institution. This fact caused his location in Atlanta. While engaged in the able and faithful discharge of his duties, as a member of the faculty of the Atlanta Medical College, he found time for an extensive and profitable practice.

The same public spirit that characterized Dr. Powell as a citizen of Sparta, distinguished him in Atlanta. He was so constituted that his mind was constantly conceiving new methods by which to advance the interests of this city and section.

He was one of the first to suggest and urge the building of a railroad from the Central Railroad via Eatonton to the Gate City, a charter for which was soon secured. He was also the first citizen who proposed the scheme of a canal for bringing the waters of the Chattahoochee river through Atlanta, which has been realized by bringing the water supply to the city. He achieved much by his voice and pen for the varied interests of his home, comprising its hotels, churches, banks, schools, railroads, manufactories, etc.

One of his grandest conceptions, having in its scope a great and splendid charity, worthy of his head and heart, was the establishment of a Home for Invalid Ladies. It was in the year 1860 that he conceived this object, and having received the sanction of wise and good men, to whom his plan was submitted, he went to work by practical, though original methods to secure the necessary grounds and construct the required buildings. While a detailed account of his efforts to make this enterprise a success would be interesting, the limited space of this sketch forbids further allusion to the subject, except to say that this grand scheme of this philanthropist was defeated only by the fortunes of war, which destroyed, with ruthless hand, all that he built with the noble hand of charity.

Dr. Powell was a distinguished lecturer, having delivered addresses of great literary merit and power. Among these were the following themes, which he treated with great ability and eloquence: "Woman, as Daughter, Wife and Mother;" "Woman as Contrasted with Man, Physically and Spiritually;" "Achievements of Christianity;" "The Charm and Power of Music;" "The Ministry, or Power of Silence;" "Southern Institutions;" "Parlor Literature."

His lectures on "Music," and "Independent Thought," elicited universal praise. His lecture on "The True Physician" was awarded a prize of seventy-five dollars by the Medical

Association of Georgia, as a model of literary beauty and excellence. He generously declined to accept the prize.

The doctor was no less distinguished as a writer, having contributed many valuable papers to the medical literature of this country, which have been published in pamphlet and book form, as well as in the medical journals of the country.

As the founder and senior editor of "THE SOUTHERN MEDICAL RECORD," he has accomplished much for the advance of science and the good of mankind. For nearly twenty-six years this magazine has gone out to the medical profession of the United States, freighted with the reports of the discoveries in the causes and treatment of human maladies.

Dr. Powell was a zealous member of the American Medical Association, and served one term as president of the American Editors' Association. He was a life-long member of the Medical Association of Georgia. As a member of the Atlanta Board of Education he had been most efficient, having from the earliest establishment of her public school system supported it with all his influence.

Dr. Powell, having, in 1866, severed his connection with the Atlanta Medical College, was urged by many of his professional friends and other leading men, to establish a new medical school in Atlanta. This he declined to do until 1879, when he deemed the time had come for such a movement. In connection with some of his professional friends, he decided to carry out this enterprise, and with indomitable will went about to accomplish this enterprise.

In the presence of many obstacles, a board of trustees was organized, comprising prominent merchants, ministers, and statesmen, with Governor Stephens at the head. A charter was secured and the organization was duly effected. The trustees went promptly to work, electing Dr. Powell president, and a faculty of eminent physicians as teachers in the new school, which was named, "The Southern Medical College."

A building committee, consisting of Dr. Powell and two other members of the faculty, was appointed to purchase a suitable lot on which to erect a large and substantial college building. To fully comprehend the situation, it should be known that there was not then a dollar at the control of the committee. The whole matter was placed under the management of Dr. Powell, to whose financial ability all who were interested looked with hope and expectation. He being regarded invincible the faculty and friends of the new college were by no means doubtful as to the ultimate success. It was in June that the committee was appointed, and to the astonishment of the community, the corner stone of a spacious and splendid college building was laid on the eighth day of the following July by the Masonic brotherhood.

On this occasion he delivered an address which was regarded as one of the greatest efforts of his life, inspiring his large, wealthy and intelligent audience with a deep and lasting sympathy for the Southern Medical College, laying then, in the hearts of the people, the foundation of that generous patronage which this institution has since merited and enjoyed.

In the following October, the college building being nearly completed, and being well equipped, was thrown open for sixty-four matriculates, probably a greater number than had ever before attended the opening session of a medical college in this country.

At the second session, the matriculates numbered more than one hundred, and the building was then thoroughly fitted up with museum, plates, models, laboratory, dispensary and a dissecting room, having every modern appliance. The herculean task of building and establishing the Southern Medical College having been performed with an excellence and promptitude which none but Dr. Powell could have done, he conceived the purpose of creating a hospital in connection with the institution. This he deemed a work of great importance to the thorough medical education of the student, and therefore went to work with his customary wisdom and energy to accomplish this plan.

He suggested his wishes to his many lady friends in Atlanta, who promptly organized "The Ladies Hospital Association," and through the medium of fairs, voluntary subscriptions, etc., raised an amount which secured the site of what was called "The Ivy Street Hospital."

Subsequent efforts of the Ladies' Hospital Association secured funds for building, furnishing and otherwise improving the hospital, accommodating about two hundred patients.

From the date of opening the hospital until the summer of 1891, it was not only a most important auxiliary of the Southern Medical College, but a means of great convenience and economy to the city of Atlanta, by providing a home and medical treatment for her sick and indigent citizens, and to the afflicted generally who have applied for a home under its roof.

It was in the midst of his labors, when Dr. Powell was at his best, that a very gratifying incident occurred. At the opening exercises of the Southern Medical College, on October 6, 1886, the speaker arose from his seat in the auditory and made the following remarks, while Dr. Powell remained standing within the railing:

"MR. PRESIDENT: Allow me to interrupt your course of proceedings with a few words. Trusting that this casket which I hold in my hand may not prove a Pandora's box, nor that the bearer may be feared, as was the case with the Greeks of old bearing presents, no apprehension should be felt by you. I would say to the ladies and gentlemen in attendance that my predecessor in the chair of surgery in retiring from the Southern Medical College, carried with him such pleasing recollections of his associations with our worthy president, that he has sent back a kind memento of personal and professional consideration for him during their relations in this institution.

"I am pleased to inform you, Dr. Powell, that it devolves upon me to represent the faculty in conveying this evidence of high appreciation by our former colleague whose heart turns to you with the affection of a son for his father. This testimonial has been prepared in the most approved style of art, with the expressive motto, *virtus in arduis*, indicating that energetic persistence in the performance of duty which has always characterized your undertakings, and which has always been crowned with such eminent success.

"Among the trophies of your successful career stand prominently the commodious college building in which we are assembled to-day, and the growing prosperity of this institution, which was inaugurated here six years ago. Those qualities of head and heart indicated by *virtus in arduis* have been worthily exemplified by your past achievements, and we trust, as you go forward and upward in higher and greater attainments, that *crescit eundo*, may be inscribed on this testimonial to your fair fame and good name, which is now delivered to you to be worn as a badge of honorable distinction."

This beautiful testimonial, fashioned in the most chaste style, being handed to Dr. Powell, he responded under evident emotion, indicating that he was most profoundly impressed by the reception of this evidence of cordial regard from his friend and former colleague, whose home, California, where he found the nugget of gold which was sent to the faculty with the request that the nugget be made into a medal and presented to President Thomas S. Powell.

Among other important remarks, President Powell presented the following striking points in his happy reply:

"It has often been surprising to me that a thorough pessimist could find birth and sustain existence in a world like this of ours.

"Despite the dark sides of this life, it has so many bright and tender tones of coloring, we often catch glimpses and inspirations of Eden-like beauty that cheer and buoy up our spirits amid the darkest gloom, and make our hearts open to catch the refreshing dews of man's love to man, as a flower unfolds its petals to the cool kisses of a summer night.

"It is needless to say, my dear doctor, that the presentation of this memento of artistic and beautiful workmanship by Dr. Thad Johnson, and the faculty of this institution, is to me, a glimpse of one of the brightest and most refreshing incidents I could meet with in the later years of my professional life. Its memory will be to me grateful and everlasting, and this medal, the eloquent token of fraternal affection and appreciation, shall be valued as one of my most valued possessions.

"Of the flattering estimates expressed to-day of my personal ability, shown in my career, I know not how to thank those noble hearted professional friends, especially, as I so deeply feel how little I have done commensurate with what I trust, is a worthy ambition. It never has been difficult for me to have faith in a good cause. If an enterprise has the approval of Heaven, of noble men and women, and is pushed forward with zeal, energy, perseverance, and all legitimate methods, I can see no such word as 'fail' written in the undertaking. If

I know I am right, I never fear obstacles; if they are small and insignificant, they are the more easily removed; if they are large, it only requires greater and grander efforts to remove them.

"Rev. Sam Jones has said that he would not mind being swallowed by a whale, but he hated to be nibbled by minnows. I think the Rev. Sam is right, but we must remember he has not much flesh to spare. Now if I had to undergo either ordeal, unless I should first lose some of my present avoirdupois, I think it would require considerable of a whale to swallow me up.

"I am proud of the motto engraved on this medal, and I would especially urge the young men present, and more especially those who have chosen the medical profession, to take 'Virtus in arduis' for a watchword, and to remember that, under God, 'All things come to those who watch and wait.'"

On account of the rapid growth of the institution, it was found necessary to erect a new building for the accomodation of the students, and accordingly a magnificent brick edifice was erected a few years ago, directly in front of the Grady hospital. A separate building for the dental department was subsequently erected. The college is one of the best organized and most thoroughly equipped in the South; and, its faculty, composed of the leading practitioners in Atlanta, is one of the ablest in the country. The service that Dr. Powell has rendered the community and the medical profession in the establishment of this institution, cannot be estimated.

In life it was a source to him of pride and delight, and in death it will prove a lasting monument to his memory. Dr. Powell not only contributed largely in the purchase of bonds for the new college, but enlisted the interest of the members of the faculty, who devote their yearly receipts towards liquidating a debt, and placing the building and college on a firm basis, when the Southern Medical College, managed as ever by trustees, will be perpetually an educational institution, and will be a credit, an honor, and profit, to those who have worked so hard for its growth.

He expressed some of these ideas in a masterly speech delivered on the occasion of the laying of the corner stone of the present building.

He said moreover: "We propose to have the Southern Medical College fully equipped for all purposes, and with the curriculum of its four departments second to no other. We will first build upon a solid basis, then proceed slowly and steadily to develop our plans, and finally establish the University complete in its every department and up to a high standard that will make it worthy the commendation of the public and the patronage of the professions it will represent."

Dr. Powell's own character may well be illustrated by a sentence he uttered on this occasion: "The very thought of what a man, under God, may do for himself and his brother in humanity inspires and uplifts the soul like the the echo of an angel's song."

Dr. Powell was one of the most industrious men who ever lived in Georgia. He was never idle in mind or body, but was constantly engaged conceiving and executing plans for the general welfare.

In reviewing the history of Dr. Powell, it is difficult to decide whether to admire more his characteristics of mind and heart, or the splendid details of his life-work. The nobility of his heart is discovered in his broad charities, his splendid patriotism and his devotion to truth and morality. The powers of his mind are apparent in his writings, his addresses and the institutions he has originated and established.

He was never known to falter by the way, nor to succumb to rival or opposing influences. In all things, and under all circumstances, he proved himself a true man, and has made a record of which any one should feel proud.

As the executive head of a famous institution of learning, Dr. Powell equipped hundreds of young physicians for the practice of their profession, besides devoting a large part of his own time to alleviating the ills of humanity. His name became a household word in every part of the country, and no man was more highly honored for his professional attainments, or more sincerely beloved for his true nobility of character. Dr. Powell always occupied a high seat in the confidence and esteem of his medical brethren, and this was due to the fact that he was not only a skillful practitioner, but that in spite of the seductions of a large and growing patronage, never bartered his principles for gain, or overlooked for a single instant the moral ethics of the profession.

In concluding this outline of the characteristic features of this ideal member of the medical profession, I wish to urge upon the young men who are now entering upon their field of duty, to imitate the example which has been set before them. With an experience of half a century in the practical work of the physician—more than a quarter of a century in teaching medicine to others—Dr. Powell has exemplified the highest attributes of devotion to the welfare of his patients, and of zealous interest in the advancement of his boys, as he was pleased to call the students.

His noble bearing as a gentleman, his sincere appreciation of ethical deportment towards others, his great success in his special department of practice, and his great administrative ability as president of the Southern Medical College, entitle his memory to most profound respect and consideration from those who have gone forth from this college in former years, and more especially from you who have recently been under his instruction in obstetrics and diseases of women. I commend to you this representative of the highest type of the medical profession as a model for imitation, and deserving to be cherished in memory throughout your professional career.

